# Un corps étranger trachéo bronchique inhabituel “l’épingle à foulard”: présentation et gestion

**DOI:** 10.11604/pamj.2015.21.327.5350

**Published:** 2015-08-31

**Authors:** Rachid Marouf, Ihssan Alloubi

**Affiliations:** 1 Service de Chirurgie Thoracique, CHU Mohammed VI, Oujda, Maroc

**Keywords:** Corps étranger, épingle à foulard, bronchoscopie, bronches, Foreign body, Scarf pin, bronchoscopy, bronchi

## Abstract

L'inhalation accidentelle du corps étranger est rare chez les adultes et les adolescents. L’épingle à foulard est un corps étranger particulier de plus en plus fréquent chez les femmes qui portent le voile. Le but de notre travail est de décrire les particularités liées à l'inhalation de cet objet éguisé à conséquences lourdes, et la nécessité d'un programme de sensibilisation et d’éducation. Dix jeunes patientes, toutes voilées, ont été hospitalisées au service de chirurgie thoracique du CHU Mohammed VI d'Oujda entre janvier 2010 et juillet 2014 pour inhalation d’épingle à foulard. La moyenne d’âge a été de 15 ans. L'inhalation a été accidentelle dans tous les cas, alors que les patientes s'initiaient au port du voile. Le syndrome de pénétration a été retrouvé dans tous les cas. L'examen clinique a été normal chez toutes les patientes. La radiographie thoracique a montré le corps étranger sous forme d'une opacité linéaire, localisée au niveau de la trachée dans 3 cas, à droite dans 4 cas, gauche dans 3 cas. La bronchoscopie rigide a réussi à extraire l’épingle dans 8 cas. L’épingle a été rejetée spontanément dans un cas et une patiente a dû être opérée. L’épingle à foulard est un corps étranger particulier de plus en plus fréquent chez les femmes qui portent le voile islamique, il faut souligner la nécessité d'un programme d’éducation de la santé envers cette population. La bronchoscopie rigide reste le principal outil de récupération de ces corps étrangers inhalés.

## Introduction

L′inhalation d′un corps étranger (CE) est rare chez l′adulte, bien que plus fréquente chez l′enfant. Le type de CE varie considérablement selon les habitudes alimentaires et l′éducation des populations étudiées. Au Maroc, les CE métalliques sont fréquents, en particulier les épingles droites utilisées pour attacher le foulard chez les femmes porteuses de viole. L'inhalation accidentelle de ces épingles aboutit le plus souvent à leur localisation dans l'arbre trachéobronchique. L'extraction de ces types de CE pointus et potentiellement pénétrants est un défi qui nécessite une attention particulière. La mise au point de ce problème critique permettra sa prévention et sa prise en charge.

## Méthodes

Dix jeunes patientes, toutes voilées, ont été hospitalisées au service de chirurgie thoracique du CHU Mohammed VI d′Oujda entre janvier 2010 et juillet 2014 pour inhalation d’épingle à foulard. La moyenne d’âge était de 15 ans avec des extrêmes de 13 à 27 ans. Aucun facteur favorisant: troubles de déglutition, maladie neurologique, neuromusculaire ou métabolique n'a été retrouvé. Toutes les patientes ont été soumises à un interrogatoire précis analysant l'ensemble des symptômes évocateurs d'inhalation de CE: toux, étouffement, raucité de voix, cyanose, hémoptysie, dyspnée et douleurs thoraciques. L'analyse de la cause, du temps écoulé depuis l'inhalation, a été aussi obtenue. Toutes les patientes avaient un CE radio-opaque à la radiographie thoracique. La bronchoscopie rigide (BR), réalisée chez 9 patientes, a constitué la procédure thérapeutique essentielle. Un contrôle radiologique a été systématiquement réalisé en post-endoscopie et la majorité des patientes ont pu quitter le service le jour même ou le jour suivant.

## Résultats

**Figure 1:**
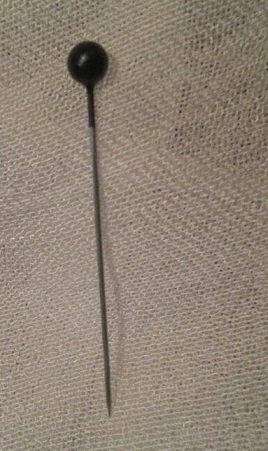
Epingles métalliques longues de 2 à 3cm avec une tête en perle de plastique radio transparente, et une extrémité métallique pointue

Le CE inhalé était une épingle à foulard dans tous les cas. Dans un cas, il s'agissait de l'inhalation simultanée de deux épingles. L’épingle à foulard était une épingle métallique longue de 2 à 3cm avec bout métallique pointu et bout capuchonné en plastique. Les patientes mettaient l’épingle entre leurs lèvres ou leurs dents avant de fixer leurs foulards (Figure 1). L'inhalation d’épingle a été survenue de façon accidentelle dans tous les cas en parlant, riant ou en toussant simultanément. Le délai moyen entre inhalation de l’épingle à foulard et l'admission au service était de de quelques heures à 4 jours. Le symptôme le plus fréquemment rapporté par les patientes était le syndrome de pénétration, défini comme des quintes de toux explosives et soudaines rapporté dans tous les cas. Une hémoptysie et un syndrome bronchique ont été rapportés dans 4 des cas chacun. L'examen clinique a été sans particularité dans tous les cas.

**Figure 2:**
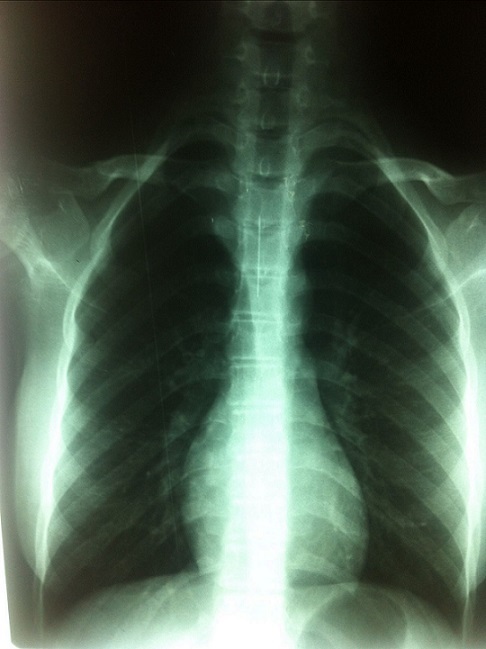
Radiographie thoracique de face montrant une épingle à foulard au niveau du 1/3 inférieur de la trachée

**Figure 3:**
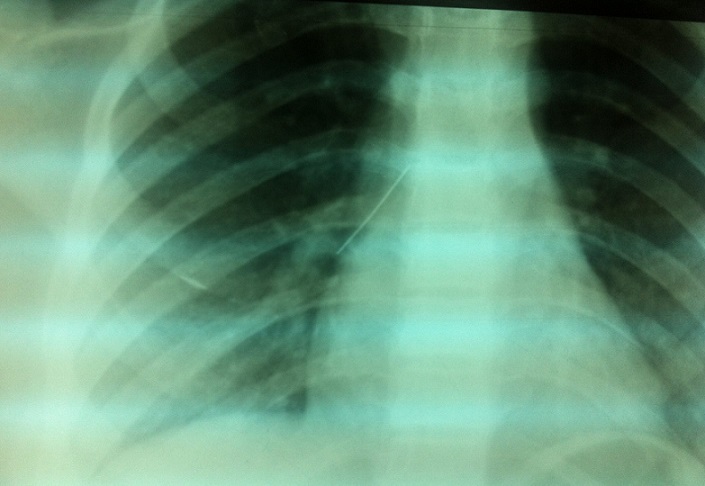
Radiographie thoracique de face montrant une épingle à foulard au niveau du tronc intermédiaire

**Figure 4:**
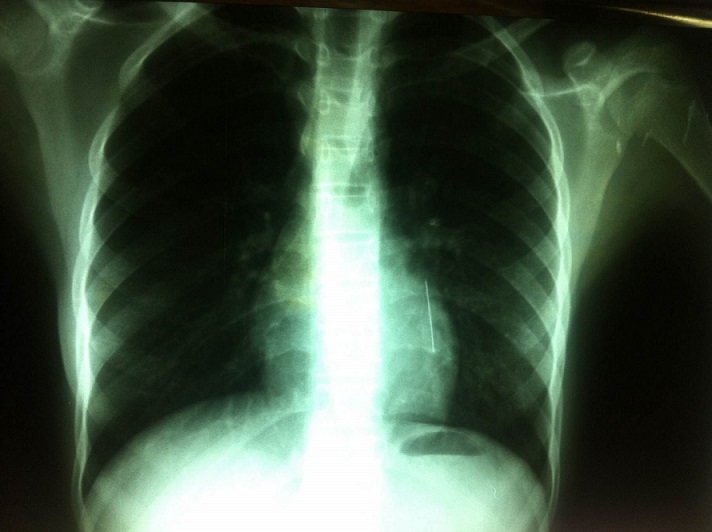
Radiographie thoracique de face montrant une épingle à foulard au niveau du segment paracardiaque gauche

**Figure 5:**
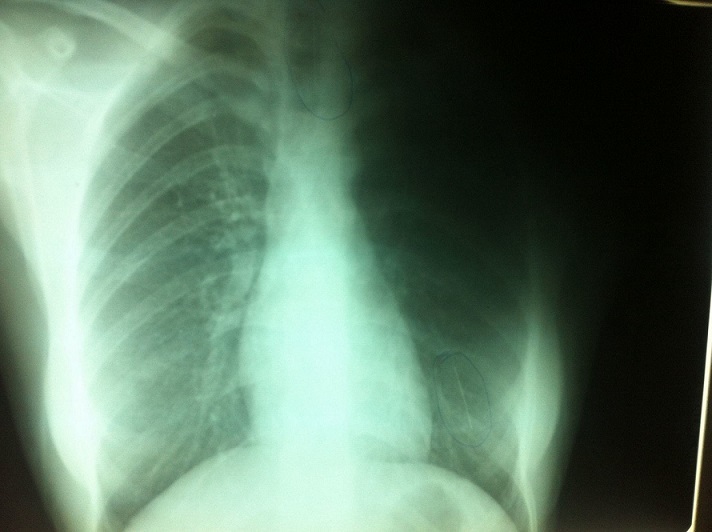
Radiographie thoracique de face montrant deux épingles à foulard: une au niveau de la trachée et l'autre au niveau du segment paracardiaque gauche

La radiographie thoracique face et profil a identifié le CE sous forme d'une opacité linéaire radio-opaque, sans anomalie parenchymateuse ou pleurale associée, dans tous les cas. Elle a localisé le CE à droite dans 4 cas, à gauche dans 3 cas, et dans la trachée dans 3 cas (Figure 2, Figure 3, Figure 4, Figure 5). La BR, réalisée chez 9 patientes, a permis de visualiser le CE au niveau de la trachée dans 3 cas, la bronche principale gauche dans un cas, la lobaire inférieure gauche dans 2 cas, le tronc intermédiaire dans deux cas et la lobaire inférieure droite dans deux cas. Le CE n'a pas été visualisé dans un cas. Des granulomes inflammatoires étaient présents dans 2 cas et des sécrétions muco purulentes dans 2 cas. La manoeuvre d'extraction par BR, a été réussie en première intention dans 8 cas, non réussie dans un cas avec recourt à une thoracotomie gauche avec extraction chirurgicale. Dans un autre cas, le CE a été rejetée spontanément. Un contrôle endoscopique après extraction n'a pas montré de réaction ou de lésion de la muqueuse bronchique. Le contrôle radiologique a montré une bonne évolution dans tous les cas. Un traitement médical comprenant une antibiothérapie et une corticothérapie de courte durée a été associé dans 5 cas.

## Discussion

Les corps étrangers (CE) intra bronchiques par inhalation accidentelle sont particulièrement fréquents dans la première enfance avec une prédominance masculine. Chez l'adulte, l'inhalation de CE est rare. Elle est plutôt observée après la soixantaine du fait de l'incidence des fausses routes [1, 2]. Les CE communs incluent les matières organiques tel que les aliments, les cacahouètes, le maïs, les grains de café… et les objets inorganiques et métalliques: pièces de monnaie, prothèses dentaires, Morceaux de jouets, de stylos ou de bijoux… [2].

Cependant, les conditions socioculturelles et régionales peuvent influencer la nature du CE inhalé. En effet, dans les pays islamiques, les femmes et les jeunes filles utilisent largement des épingles droites pour fixer leurs foulards. Il s'agit d’épingles métalliques longues de 2 à 3cm avec une tête en perle de plastique radio transparente, et une extrémité métallique pointue [1, 3]. L′inhalation d’épingle à foulard est constamment rapportée chez des jeunes adolescentes, qui s'initient au port du voile, qui sont habituellement moins attentives et moins habiles que les femmes adultes à la maîtrise et l'ajustement du foulard [1, 3].

Il s′agit d′une pratique assez complexe. En effet, la femme maintien la tige de l′épingle entre les dents alors que les deux mains sont utilisées pour régler le foulard sur la tête. L′aspiration accidentelle se produit habituellement pendant la parole, la toux, le rire, ou en prenant une inspiration profonde tandis que la tête est inclinée vers l'arrière [3].

Si le CE pénètre dans l′arbre bronchique, la bronche souche droite est le plus souvent en cause, en raison de son obliquité (les angles bronchiques avec l′axe trachéal sont similaires chez l′enfant et chez l′adulte, 30° à droite, 45° à gauche) et de son calibre légèrement supérieur à celui de la bronche souche gauche, Pourtant, ce n’était pas le cas pour d'autres séries [1, 2]. Les patientes présentent le moment de l′inhalation un syndrome de pénétration, puis deviennent asymptomatiques après une période de toux intense témoignant de la nature non asphyxiante de ce type de CE. Le risque majeur est la mobilisation intempestive de cet objet acéré [4].

En raison de la nature métallique et radio-opaque de ce CE, les examens radiologiques de routine doivent inclure une radiographie cervicothoracique face et profil et un abdomen sans préparation afin d’écarter la possibilité d'ingestion du CE [2]. La fibroscopie bronchique souple (FBS) est couramment utilisé pour diagnostiquer et récupérer les corps étrangers trachéo-bronchiques chez les enfants et les adultes avec un taux de réussite élevé. Toutefois, en cas d'inhalation d′épingle à foulard, la bronchoscopie rigide (BR) est considéré comme la procédure standard pour l′extraction et FBS a été rarement utilisée [5]. Il existe de nombreuses études sur l'utilité de FBS sous une sédation consciente et anesthésie locale pour la récupération des épingles à foulard. Shabb et al. (Shabb 1996) ont rapporté 5 cas de broches atmosphériques qui ont été récupérés avec succès par FBS sous anesthésie générale [6].

Dans une étude rétrospective, Gokirmak et al. (Gokirmak 2002) un rapport sur l′utilisation de FBS sous anesthésie locale pour la récupération de broches inhalées dans 11 cas avec un taux de 73% de réussite [7]. Dans la série de N. Zeghba et al, sur une série de 26 patientes, la FBS a réussit à extraire le CE dans 80s% des cas [8].

Certes, une complication potentielle de l′extraction du CE avec FBS sous sédation consciente et anesthésie locale est le risque d′endommager l′arbre trachéobronchique ou le larynx lors de la récupération de l′épingle à foulard. Une autre complication possible est le risque de perdre la broche dans la gorge. La broche est ensuite rapidement avalé et s′installe dans l'estomac [3]. La BR permet un grand accès aux voies aériennes trachéo bronchiques assurant une oxygénation correcte et un passage facile du fibroscope et des pinces, ce qui permet une extraction rapide, efficace et sécurisée de l’épingle dont l'extrémité pointue risque de s′incruster profondément dans la muqueuse [1, 9]. Le taux de thoracotomie rapporté dans les séries varie entre 1,6 et 18% [2, 5, 6]. Dans la série d'Al-Ali et al. [10], il est de 6%. Le recours à la thoracotomie est relié à la localisation distale de l’épingle, le retard de consultation après inhalation et la formation de granulomes autour de l’épingle inhalée.

## Conclusion

L'utilisation de l’épingle à foulard est une pratique culturelle qui porte de graves risques sur la santé. La prévention passe par l’éducation et la sensibilisation de la communauté des adolescentes et des jeunes adultes qui s'initient au port du voile, par des affiches et des autocollants spécifiant les dangers à l'utilisation imprudente de ces épingles, et aussi par la réglementation de la commercialisation de ces objets.
